# A Pilot Study: Attention Deficit Hyperactivity Disorder, Sensation Seeking, and Pubertal Changes

**DOI:** 10.1100/tsw.2006.129

**Published:** 2006-06-12

**Authors:** Catherine A. Martin, Greg Guenthner, Christopher Bingcang, W. Jackson Smith, Thomas Curry, Hatim A. Omar, Mary Kay Raynes, Thomas H. Kelly

**Affiliations:** ^1^ Department of Psychiatry, University of Kentucky College of Medicine, Lexington, KY 40536, USA; ^2^ Department of Pediatrics, University of Kentucky College of Medicine, Lexington, KY 40536, USA; ^3^ Department of Obstetrics and Gynecology, University of Kentucky College of Medicine, Lexington, KY 40536, USA; ^4^ Department of Behavioral Science, University of Kentucky College of Medicine, Lexington, KY 40536, USA; ^5^ College of Nursing, University of Kentucky College of Medicine, Lexington, KY 40536, USA

**Keywords:** puberty, sensation seeking, Attention Deficit Hyperactivity Disorder, testosterone, DHEAS, sex, United States

## Abstract

This study was designed to examine the relationship of pubertal changes and sensation seeking (SS) in adolescents with Attention Deficit Hyperactivity Disorder (ADHD). Patients with current or past histories of uncomplicated stimulant medication use for ADHD between the ages of 11 and 15 (13 ± 1.5) were recruited from a Child Psychiatry and a General Pediatric Clinic. SS was measured using the SS Scale for Children. Pubertal development was measured using Tanner staging, free testosterone, and DHEAS. Subjects and their parent were interviewed with the Diagnostic Interview Schedule for Children (DISC). SS total score was correlated with Tanner stage, free testosterone, and DHEAS (*p* ≤ 0.01). The combined parent and child reports of symptoms of Oppositional Defiant Disorder from the DISC were inversely related to age (*p* ≤ 0.05). Understanding SS in ADHD adolescents as they move through puberty will aid clinicians in monitoring ADHD adolescents and their trajectory into high-risk behaviors.

## INTRODUCTION

Sensation seeking (SS) has been correlated with drug use and other high-risk behaviors in adolescents[1,2]. SS has been associated with Oppositional Defiant Disorder (ODD) and Conduct Disorder (CD) symptoms, but not Attention Deficit Hyperactivity Disorder (ADHD) symptoms in early- and mid-adolescent patients[3]. While the characterization of SS in an ADHD population has received little consideration, it may be particularly important as the ADHD adolescent enters puberty and the phase of life where drug abuse risk increases[4,5].

The association of age and SS is well described in adolescents[6] and adults[7]. SS has also been associated with pubertal stage[8,9,10] and sex steroids, specifically testosterone levels in adult males[11,12] and estrogen levels in college females and males[12]. The Novelty-Seeking subscale of

Cloninger's Tridimensional Personality Questionnaire and a clinical assessment of SS based on the Zuckerman's SS scale have been associated with testosterone levels in adult males[13,14]. Elevated scores on the Monotony Avoidance scale from the Karolinska Scale of Personality have been associated with high testosterone levels in delinquents[15].

The aim of this study is to investigate the relationships of SS and Disruptive Disorder (DD) symptoms with age, pubertal changes, free testosterone, and DHEAS in an ADHD population. Characterization of SS in ADHD adolescents as they move through puberty will add to the limited understanding of ADHD adolescents and their trajectory into high-risk behaviors.

## METHODS

Participants between the ages of 11 and 15 years (13 ± 1.5) were recruited from the Child Psychiatry Clinic and two pediatric clinics. The Child Psychiatry Clinic focused on, but did not provide care exclusively to, youth with DD (in particular ADHD). Patients with current or past histories of uncomplicated stimulant medication use for ADHD, who were not using other psychotropic medications currently and had no significant medical or psychiatric disorders other than ADHD, were recruited from these clinics. Parental consent and assent from the youth were obtained from all participants.

Following attempts to recruit an equal number of males and females at various Tanner stages, the final sample consisted of 13 females and 11 males. The following Tanner stages were represented: one in Stage 1, eleven in Stage 2, five in Stage 3, two in Stage 4, and five in stage 5. Four were black and one subject reported being biracial. The drug screens that occurred on the days the sex steroids were assayed were negative.

## Clinical Screen

### *Questionnaire Items*

- **Sensation Seeking***:* SS was measured using the Russo's Sensation Seeking Scales for Children (SSSC). This scale, a modification of Zuckerman's SS scale, measures the respondents' need to experience varied, novel, and complex sensations[6].
- **Diagnostic Interview Schedule for Children-Revised (DISC-R), Parent and Child Version***:* DISC-R identifies psychiatric disorders and psychological symptoms in children and adolescents[16]. Symptoms from both the child and parent interviews were summed into the following four scales: hyperactivity, inattention, ODD, and CD.
- **Pubertal Stage**: Sexual maturation staging was performed by physical exam using Tanner classification.
- **Blood Samples for Hormone Measures***:* Blood samples were collected on two separate days and free testosterone and DHEAS were assayed. The average of the two assays was entered into the analysis. Because of the small number of subjects, hormones with cyclical variation (e.g., estradiol, progesterone) were not included in the analysis. Ten cc of blood were drawn into red top vacutainer tubes, centrifuged, and transported on ice to the Reproductive Endocrinology Laboratory at the University of Kentucky. Serum was frozen until assayed for free testosterone and DHEAS by radioimmunoassay (RIA) using Coat-A-Count kits (Diagnostic Products Corp., Los Angeles, CA). These kits are direct, solid phase 125I RIA kits. Each respective hormone was assayed in the subject's serum by incubating the serum with the primary antibody per the manufacturer's recommendations. Samples were decanted, counted, and hormone concentration determined by reference to a standard curve. The assay sensitivity for each hormone are as follows: free testosterone was 0.15 pg/ml and DHEAS was 1.1 μg/day. The intra- and interassay coefficients of variation were routinely between 6 and 9% and 10 and14%, respectively.

## Laboratory Protocol

Prior to the blood assays, parents were instructed on the application of EMLA 1 h before coming to the laboratory to assist in decreasing stress related to the blood draw. The DISC-R, the physical exam for Tanner staging, and urine drug screen occurred at this time.

All subjects arrived at a General Clinical Research Center at 8:30 am on two mornings separated by 1, 2, or 3 days. They were required to fast the morning of the study and were told to withhold all medication until after the lab session was completed. On arrival, breath samples were analyzed with a hand-held alcohol Breathalyzer (Alco-Sensor, Intoximeters, Inc.). Saliva was collected and analyzed for cotinine, and a urine sample was screened for pregnancy (in females) and for the presence of amphetamines and other substances of abuse. Subjects were aware that any evidence of drug use other than their current medication would prevent their participation in the study.

## Statistical Evaluations

For SS total score and Tanner stage, t tests were performed to determine if there were significant gender differences in the scores. Spearman's rank correlation coefficients were used to determine if SS was associated with age, Tanner stage, mean free testosterone, and DHEAS both in the full sample and for each gender separately. Summation scores for DD (hyperactivity, inattention, ODD, and CD) obtained from the DISC-R also were correlated with age, Tanner stage, mean free testosterone, and DHEAS. In the case of significant correlations, a Fisher transformation was performed to compare the correlation coefficients in males and females.

## RESULTS

There was no significant difference in either SS total score or Tanner stage by sex. Table 1 presents the correlation between SS, age, Tanner stage, mean free testosterone, and DHEAS. SS total score was correlated with Tanner stage, mean free testosterone, and DHEAS and only marginally with age (Spearman's rho = 0.35; $p \leq 0.10$).

**TABLE 1**
**Spearman's Rank Correlation of Age, Tanner, Mean Free Testosterone (FT), DHEAS, SS, and ODD**

| | Age | Tanner | Mean FT | Mean DHEAS |
|---|---|---|---|---|
| SS total score | 0.35 | 0.54** | 0.55** | 0.52** |
| M (n = 11 ) | 0.33 | 0.55$^{.08}$ | 0.32 | 0.30 |
| F (n = 13 ) | 0.66* | 0.70** | 0.73** | 0.62* |

*$p < 0.05$; **$p < 0.01$.

No DISC DD symptoms were correlated with Tanner stage or pubertal hormones and only ODD showed an inverse relationship to age (Spearman's rho= –0.42; $p \leq 0.05$).

The Fisher transformation did not demonstrate statistical difference in the correlations when considering gender.

## DISCUSSION

The ADHD adolescent is understudied[17] and little is known about how pubertal changes impact ADHD. This study examines physiological pubertal measures in early- and mid-adolescent ADHD patients. One of the most robust findings is the association of SS with pubertal stage, mean free testosterone, and mean DHEAS. Martin et al.[2] documented the association of SS with pubertal changes based on parent and self-report in a similarly aged larger population from both psychiatric and pediatric sources that included ADHD and non-ADHD populations. The current study was exclusive to 11–15 year olds who had received treatment for ADHD and assessment was expanded to include Tanner staging, free testosterone, DHEAS, and the DISC. This association of SS with free testosterone and DHEAS, and pubertal stage was in contrast to the lack of association of DD symptoms with pubertal stage or sex steroids. Rowe et al.[18] reported the association between testosterone and nonaggressive CD symptoms in 9- to 15-year-old boys with deviant peers, while van Goozen et al.[19] reported higher levels of DHEAS, but not testosterone in CD prepubertal boys. Neither study examined females. Testosterone has been correlated with antisocial behavior in adult males[20]. In the current study, there was no association of DISC Parent and Child reports of DD symptoms with changes in age or puberty or sex steroids. In fact there was a decrease in ODD symptoms with age in the ADHD population.

Daitzman et al.[12] found that SS correlated with estrogen levels in college females and males. In the current study, while statistically nonsignificant, there appears to be a stronger association between SS and Tanner stage, free testosterone, and DHEAS in females than males. This observation deserves further investigation.

This study is limited by a small sample size and no control group. In addition, this was a select ADHD population who had a history of successful treatment with stimulants and limited drug-taking behavior.

Measuring SS is highly associated with high-risk behavior in adolescents and can be measured easily in a clinical setting. Increased attention should be given to this personality measure as adolescents with ADHD advance through puberty. Understanding SS in ADHD adolescents as they move through puberty will aid clinicians in better identifying, monitoring, and assisting ADHD adolescents when high-risk behaviors are beginning to emerge.

## ACKNOWLEDGMENTS

This research was supported by NIDA grants number DA 05312, 1 KO8 DA00333, and NIH grant number M01 RR02602.

## BIOSKETCHES

**Catherine A. Martin**, **MD,** Professor and Vice Chair for Research, Department of Psychiatry, Child Psychiatry Division, University of Kentucky, Lexington. Her clinical and research interests are in the areas of Attention Deficit/Hyperactivity Disorder and nicotine dependence in adolescents and adults. E-mail: cmartin@uky.edu

**Gregory E. Guenthner**, **MLIS**, research assistant in the Department of Psychiatry, College of Medicine, University of Kentucky, Lexington. E-mail: gguenth@uky.edu

**Christopher M. Bingcang**, **BS,** student in the College of Medicine at University of Kentucky, Lexington. He will graduate with MD degree in May 2007. E-mail: cmbing0@uky.edu

**W. Jackson Smith**, **MD**, Associate Professor of Pediatrics and Internal Medicine, Division of Endocrinology, University of Kentucky, Lexington. Dr. Smith's research focus is in general and adolescent endocrinology and diabetes. E-mail: jsmith1@pop.uky.edu

**Thomas E. Curry, Jr., PhD**, Professor and Vice Chair, Division of Reproductive Endocrinology, Department of Obstetrics and Gynecology, University of Kentucky, Lexington. Dr. Curry's research interests are in the area of reproductive physiology. E-mail: tecurry@uky.edu

**Hatim A. Omar, MD**, Professor of Pediatrics, Obstetrics and Gynecology and Director of the Section of Adolescent Medicine, Department of Pediatrics, University of Kentucky, Lexington. He is the recipient of the Commonwealth of Kentucky Governor's Award for Community Service and Volunteerism. He is an expert in adolescent medicine and pediatric and adolescent gynecology. E-mail: haomar2@uky.edu

**Mary Kay Rayens, PhD**, Professor of Biostatistics, Department of Nursing, University of Kentucky, Lexington. Dr. Rayens' interests include tobacco policy, women's mental health, and biostatistics. E-mail: mkrayens@email.uky.edu

**Thomas H. Kelly, PhD**, Robert Strauss Professor and Associate Director for Research, Department of Behavioral Science, and K30 Principal Investigator, University of Kentucky, Lexington. Dr. Kelly's research interests are in the area of experimental analysis of human behavior. E-mail: thkelly@pop.uky.edu
